# Wilms tumor, pleuropulmonary blastoma, and DICER1: case report and literature review

**DOI:** 10.1186/s12957-018-1469-4

**Published:** 2018-08-10

**Authors:** Olivier Abbo, Kalitha Pinnagoda, Laurent Brouchet, Bertrand Leobon, Frédérique Savagner, Isabelle Oliver, Philippe Galinier, Marie-Pierre Castex, Marlène Pasquet

**Affiliations:** 1 Pediatric Surgery Department, Children’s Hospital of Toulouse, CHU Toulouse, 330 Avenue de Grande Bretagne, 31059 Toulouse, France; 2 Thoracic Surgery Department, Hôpital Larrey, CHU Toulouse, Toulouse, France; 3 Cardiac Surgery Department, Children’s Hospital of Toulouse, CHU Toulouse, 330 Avenue de Grande Bretagne, 31059 Toulouse, France; 4 Molecular Endocrinology—Institut Fédératif de Biologie, CHU Toulouse, 330 Avenue de Grande Bretagne, 31059 Toulouse, France; 5 Endocrinology Department, Children’s Hospital of Toulouse, CHU Toulouse, 330 Avenue de Grande Bretagne, 31059 Toulouse, France; 6 Pediatric Hemato-oncology Department, Children’s Hospital of Toulouse, CHU Toulouse, 330 Avenue de Grande Bretagne, 31059 Toulouse, France

**Keywords:** DICER 1, Pleuropulmonary blastoma, Wilms tumor

## Abstract

**Background:**

Pleuroblastoma (PPB) is a rare pediatric tumor which, in 30% of cases, is associated with cystic nephroma. It has been recently linked to the DICER1 mutation as part of a predisposition syndrome for various tumors. However, if DICER 1 anomalies have been reported in patients with Wilms tumor (WT), to date, no cases of PPB, WT, and DICER1 mutations have been reported in the same patient.

**Case presentation:**

We report the case of a 3-year-old patient, initially managed for metastatic WT. During his clinical course, the diagnosis of a PPB was made after detecting the DICER1 mutation and subsequent management was therefore modified.

**Conclusion:**

This case highlights that in case of simultaneous discovery of a renal tumor and a pulmonary lesion in a child, the DICER 1 mutations should be looked for as these could help adapt management and schedule the surgical procedures.

## Background

Pleuropulmonary blastoma (PPB) is a rare pediatric tumor with around 500 cases reported [1]. Its association with cystic nephroma is classically reported and occurs in 30% of patients [2]. Moreover, it has been linked to the mutation of DICER1 as part of a predisposition syndrome for different types of tumors [3]. Wilms tumor is another well-known pediatric tumor with an incidence of seven per one million cases per year [4]. Predisposition genetic syndromes and multiple mutations associated with Wilms tumor have been reported including DICER 1 anomalies. However, to date, there have been no cases of PPB, WT, and DICER1 mutations in the same patient reported in the literature.

## Case presentation

**Fig. 1:**
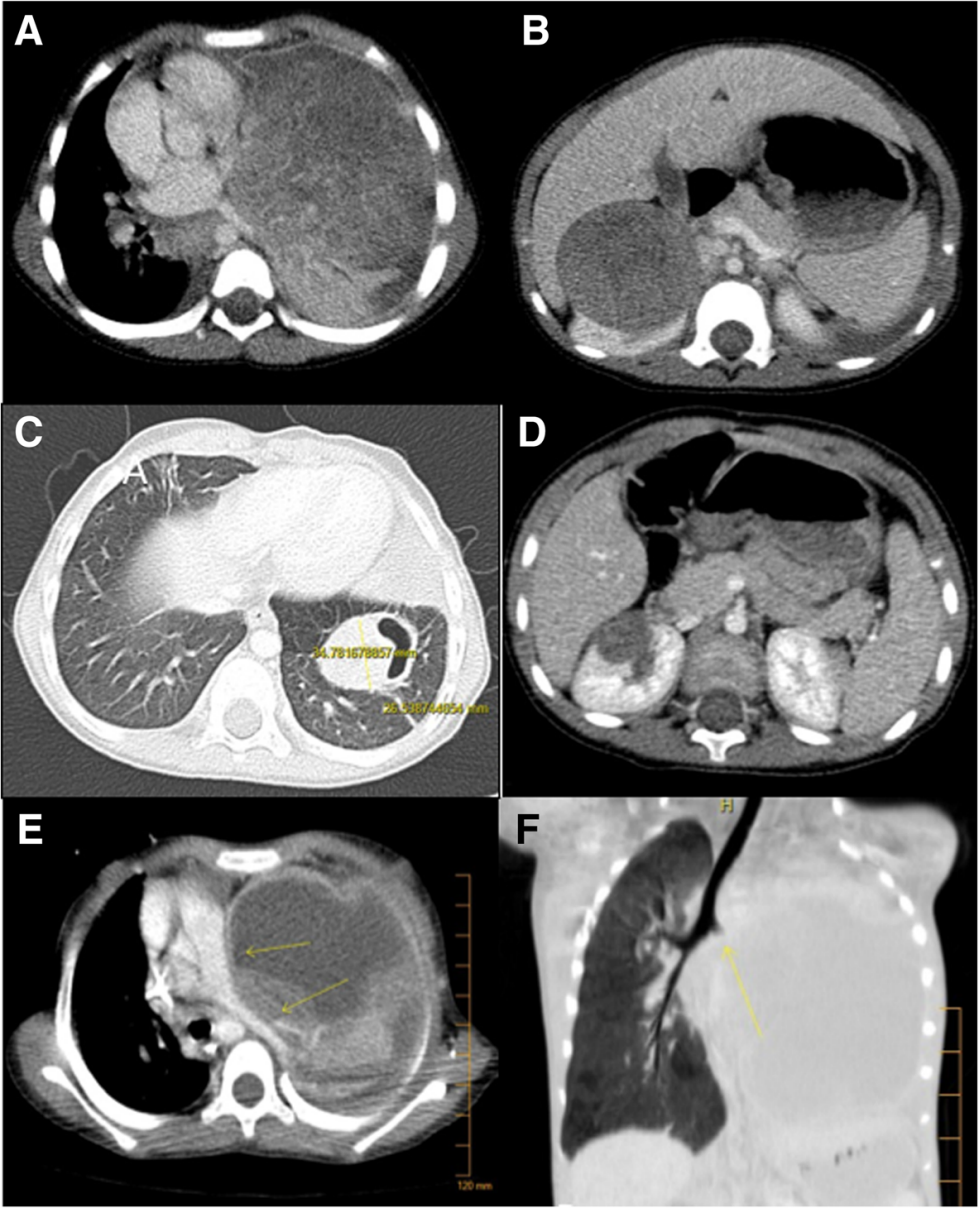
CT scan evolution through pre-operative chemotherapy. **a**, **b** Pulmonary and renal lesions at diagnosis. **c**, **d** Good response after WT chemotherapy. **e**, **f** Dramatic pre-operative evolution after modification of the chemotherapy drugs, which led to emergency pneumonectomy due to acute symptoms. Arrows indicate the narrowing pulmonary artery

A 3-year-old girl was admitted for fever and cough. She was diagnosed as having pleuresia and pleural drainage along with broad spectrum antibiotics was prescribed. Lack of improvement after a few days led to complete the work-up with a CT scan (Fig. 1a, b). The scan showed a tissular lesion of the left lower pulmonary lobe associated with a tumor of the right kidney. Lung biopsy showed blastema, without being able to distinguish whether its origin was WT or PPB despite multiple analyses by various pathological experts. Following the recommendations of the national panel of expert of both tumors, we decided to treat the patient as a metastatic WT following the International Society of Paediatric Oncology protocol (SIOP WT2001) [5]. A nephrectomy was thus performed after 6 weeks of chemotherapy (vincristine and actinomycin), with a good response in both sites (Fig. 1c, d).

**Fig. 2:**
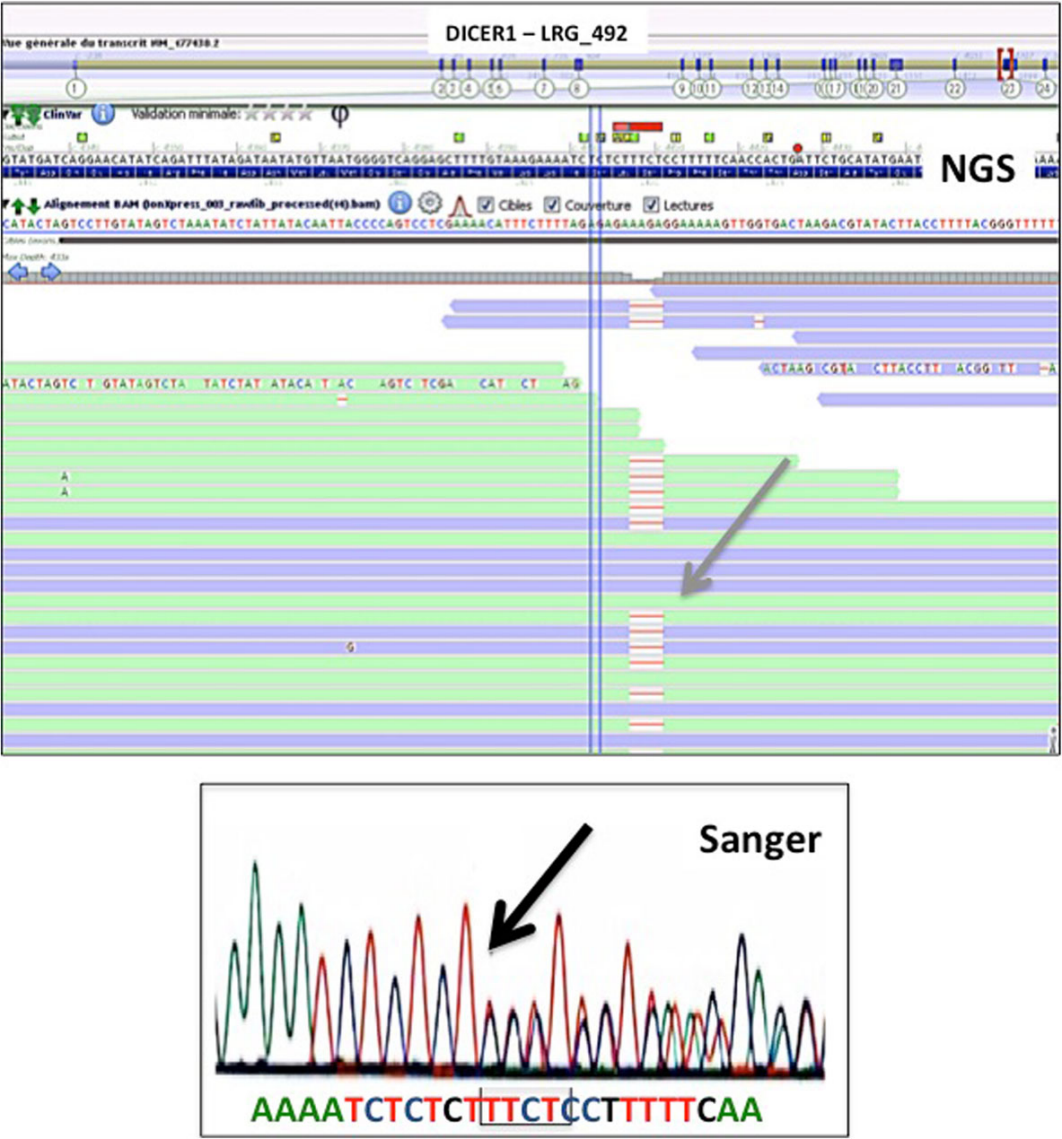
Heterozygous TTCT deletion detected by next-generation sequencing (Ion Torrent, AmpliSeq custom panel) in the exon 23 of the DICER1 gene (LRG_492, gray arrow), 320× coverage reads (Integrative Genomic Viewer software). Confirmation of the TTCT deletion by Sanger sequencing (black arrow)

Histologic analysis confirmed the diagnosis of WT with an intermediate risk (epithelial type) which was classified as a stage IV. Lung surgery was scheduled several weeks later. During this period, using high-throughput sequencing of a panel of genes involved in endocrine tumor development, we identified a heterozygous pathogenic variant in exon 23 of the DICER1 gene (LRG_492). This variant c.4407_4410del, p.Ser1470Leufs*19, leading to loss of the RNase III active site, has been previously associated with pleuropulmonary blastoma [3].The mutation was confirmed by Sanger sequencing (Fig. 2). This led to a high suspicion of associated PPB. Pre-operative workup showed increase in tumor size, and chemotherapy was modified (by adding ifosfamide and doxorubicin) to try to reduce tumor volume and vascularization. After the first course of chemotherapy, the patient developed respiratory distress due to a massive increase in tumor size, leading to mediastinal compression. It was then decided to remove the left lung (Fig. 1e, f).

A left pleuro-pneumonectomy with intra-pericardic ligature of the vessels and without cardiac assistance was performed. The early postoperative course was uneventful. The pathologist confirmed the diagnosis of type 3 PPB with R0 resection. Postoperative chemotherapy consisted of eight courses (27 weeks) of vincristine, actinomycin, and doxorubicin. After 1-year follow-up, the patient remains asymptomatic and is considered to be in remission.

## Discussion

The association of DICER 1 mutations and PPB is reported in approximatively 66% of recorded cases [1]. Among all patients with a PPB, 30% also presented with cystic nephromas [2]. Moreover, WT can be associated with DICER1 mutations [6]. However, our report is the first described case of WT, PPB, and DICER1 mutation.

Our experience underlines two aspects. First, in case of associated renal and pulmonary tumors, preoperative chemotherapy could help determine the nature of the renal lesion. In our case, the WT could be easily removed without preoperative treatment because of its anatomic localization, but we considered it as metastatic disease because of the lung tumor. The diagnosis of DICER1 mutation after the nephrectomy brought us to consider the possibility of two synchronous lesions. It would have been helpful to determine this earlier as it could have modified the surgical strategy. We could also have combined both procedures, but it seemed too difficult for the patient to cope with such an operation.

Secondly, DICER 1 mutations and WT have been reported in the genotype of tumor cells as in patients with PPB [5]. However, this is the first reported case. It confirms that DICER1 is implicated in microRNA biosynthesis in nephrogenesis and that the loss of MIRNA processing enzymes can lead to an activation of progenitor cells from stem cells, as in WT1 mutation [3, 7].

Surgical decisions were linked to the evolution of the tumor after chemotherapy. The acute lung symptoms could have been due to necrosis of the tumor inducing massive pleural effusion or by rapid tumor growth. The emergency surgical procedure was meant more to improve the child’s breathing than to actually cure her, because we thought that local invasion would mean the tumor was not resectable. Surprisingly, complete excision of the PPB was possible despite what the preoperative workup suggested.

Finally, this case should help inform parents with PPB and DICER1 mutations that WT can also occur in these circumstances and that ultrasound scan should be performed more easily in these patients to avoid late diagnosis.

## Conclusion

This yet unreported association highlights that in case of simultaneous discovery of a renal tumor and a pulmonary lesion in a child, DICER 1 mutation research could help adapt management and schedule surgery.
